# Subaortic Membrane With Mitral Chordae Rupture Mimicking Left Ventricular Outflow Tract Obstruction in Hypertrophic Cardiomyopathy

**DOI:** 10.7759/cureus.20372

**Published:** 2021-12-12

**Authors:** Mohamad Omar Choukair, Ahmad Halawi, Amal Nehmeh, Hasan Kazma

**Affiliations:** 1 Department of Cardiovascular Medicine, Lebanese University Faculty of Medical Sciences, Beirut, LBN; 2 Internal Medicine, Lebanese University Faculty of Medical Sciences, Beirut, LBN; 3 Department of Cardiology, Bahman Hospital, Beirut, LBN

**Keywords:** tte, sub aortic membrane, left ventricular outflow tract obstruction, mitral chordae rupture, hypertrophic cardiomyopathy

## Abstract

Hypertrophic cardiomyopathy (HCM) is an autosomal-dominant disorder that can lead to left ventricular outflow tract (LVOT) obstruction. Some patients present with syncope, dyspnea, chest pain, or sudden cardiac death. A subaortic membrane (SAM) is an unusual cause of ventricular outflow tract obstruction causing symptoms that can imitate HCM. It is essential to differentiate between these two entities, as it has important implications in guiding treatment and determining the type of intervention. Echocardiography is the gold standard modality for the diagnosis. In this report, we present a case of a 56-year-old man presenting with subaortic stenosis with ruptured mitral chordae misdiagnosed as HCM with SAM.

## Introduction

A subaortic membrane (SAM) is an unusual cause of left ventricular outflow tract (LVOT) obstruction and rare congenital heart disease. Hypertrophic cardiomyopathy (HCM) is an autosomal-dominant disorder that can lead to LVOT obstruction; it affects one in 500 individuals and is caused by increased wall thickness in the left ventricle not explained by abnormal loading conditions alone. Both conditions can present with syncope, dyspnea, chest pain, or sudden cardiac death. HCM with dynamic LVOT obstruction may conceal the presence of the SAM on transthoracic echocardiography (TTE) and lead to a false diagnosis. Hence, it is important to distinguish a dynamic LVOT obstruction from fixed LVOT obstruction caused by a SAM. TTE can sometimes fail to diagnose a SAM near the aortic valve; however, transesophageal echocardiography (TEE) can finely visualize perivalvular structures and help diagnose other causes of LVOT obstruction [1].

We report a case of a patient who had a SAM with dynamic LVOT obstruction with ruptured mitral chordae misdiagnosed as HCM with dynamic LVOT obstruction; the SAM was not seen initially on TTE but identified intraoperatively.

## Case presentation

**Figure 1:**
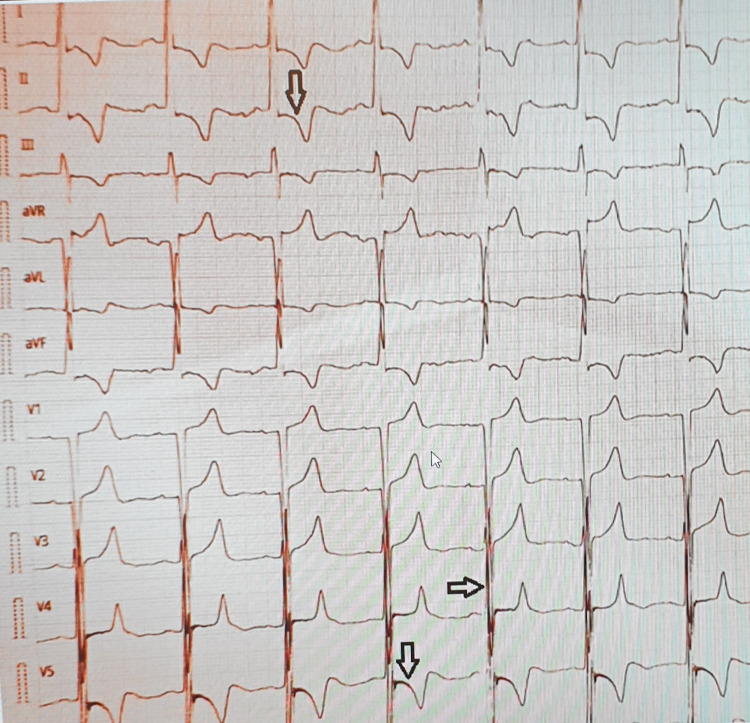
ECG of the patient showing sinus rhythm with deep T wave inversions and ST depression depicting LVH with strain pattern ECG: electrocardiogram; LVH: left ventricular hypertrophy

**Figure 2:**
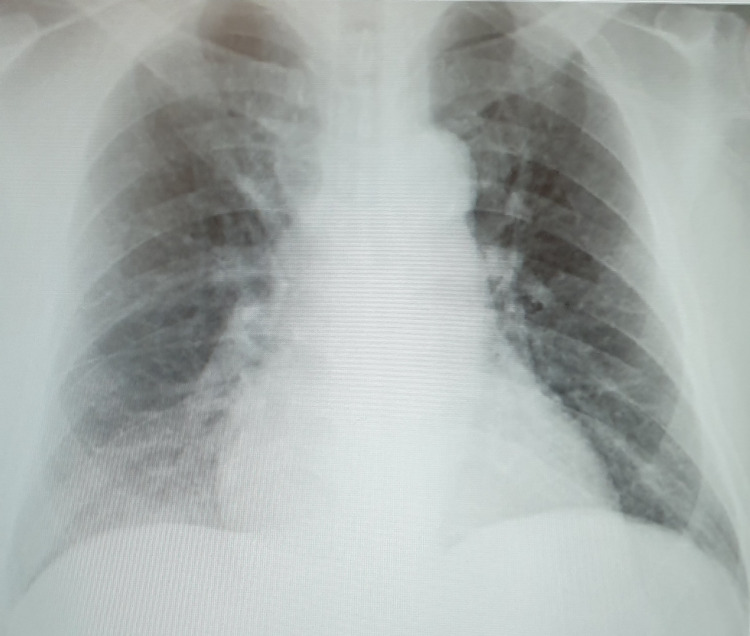
Chest X-ray shows cardiomegaly and pulmonary vascular congestion

**Figure 3:**
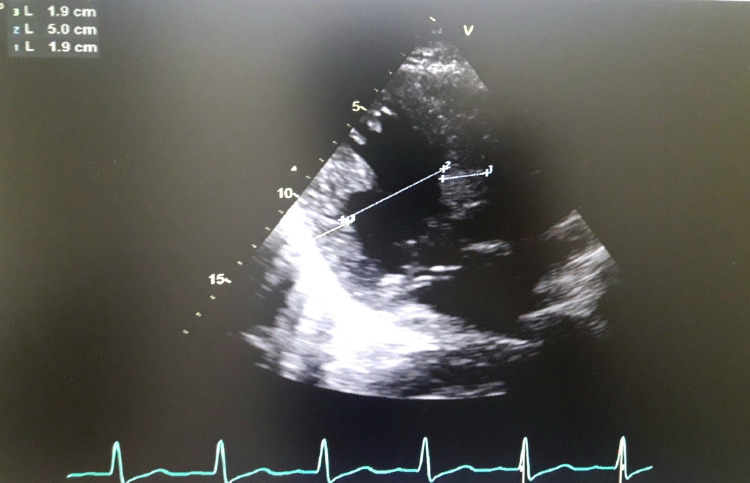
Echocardiography showing concentric hypertrophy

**Figure 4:**
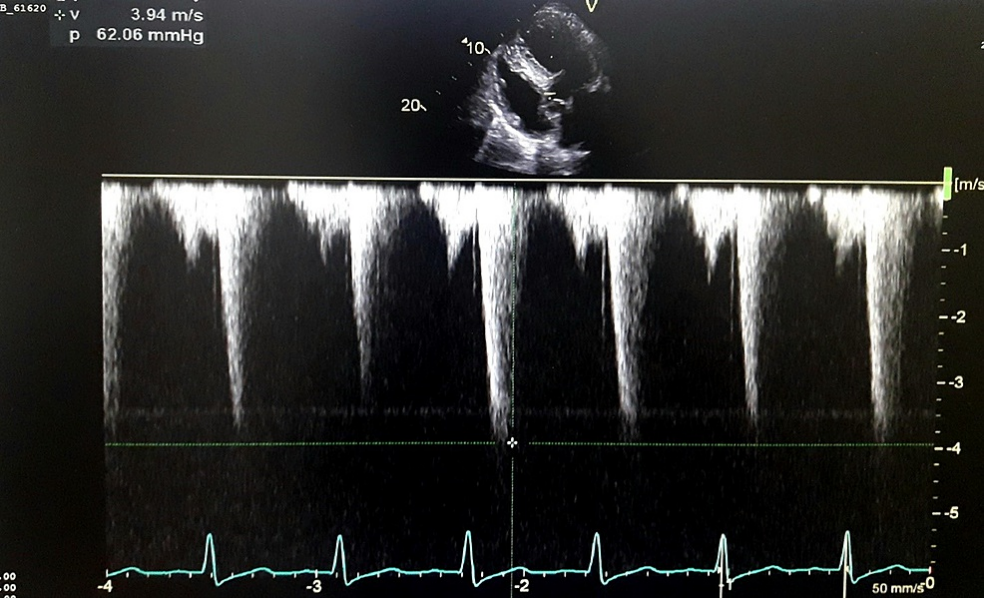
Echocardiography showing a complete SAM of the AML causing severe LVOT obstruction, with a peak gradient of 62 mmHg SAM: subaortic membrane; AML: anterior mitral leaflet; LVOT: left ventricular outflow tract

A 56-year-old male patient, a known case of diabetes and chronic obstructive pulmonary disease, presented with complaints of chest pain and shortness of breath. A 12-lead electrocardiography (Figure 1) showed sinus rhythm with deep T wave inversions and ST depression showing left ventricular hypertrophy (LVH) with strain pattern. A Chest X-ray (Figure 2) demonstrated marked cardiomegaly with pulmonary congestion; troponin was positive, and cardiac catheterization showed normal coronary arteries. TTE (Figures 3, 4) revealed concentric hypertrophy and a complete SAM of the anterior mitral leaflet (AML) causing severe LVOT obstruction, with a peak gradient of 62 mmHg. The mitral valve showed elongation of both leaflets causing SAM of the AML, with severe-grade (4/4) mitral regurgitation (MR). The aortic valve appeared structurally normal, and tricuspid with no stenosis or regurgitation.

The patient was diagnosed with HCM with SAM and was referred for surgical septal myomectomy to another institution. TEE was performed prior to the surgery and demonstrated the same result as TTE. Intraoperatively, a SAM with myxomatous mitral chordae rupture mimicking SAM with HCM was incidentally found. The surgical resection of the SAM and septal myomectomy resulted in significant symptomatic relief and lower LVOT velocities on postoperative TTE.

## Discussion

We described the case of a 56-year-old patient with clinical and diagnostic findings consistent with HCM presenting with progressive dyspnea and chest pain; the TTE showed concentric hypertrophy with dynamic LVOT obstruction, but the LVOT continuous-wave (CW) Doppler velocity waveform was not late-peaking.

The American Heart Association Task Force has suggested that TEE can help if TTE is not conclusive with limited image quality. In fact, TEE is more useful than TTE in precisely visualizing perivalvular structures and can help confirm the presence of unusual causes of severe LVOT obstruction. However, in our case, the TEE showed the same results as TTE.

A 3D echocardiography can also provide more information about the mechanism of SAM and the geometry of the LVOT in some patients. The use of cardiac MRI (CMRI) is also useful in such cases. CMRI is a sensitive modality for the identification of SAM. CMRI finely evaluates subvalvular stenosis, including the precise location and quantification of the gradient, and performs the quantitative and qualitative evaluation of LVH and ventricular function. This is needed for a cautious evaluation of alternative causes of LVOT obstruction especially when considering therapeutic interventions. Patients with LVOT can be treated medically; those who remain symptomatic with the New York Heart Association (NYHA) class III-IV with LVOT gradient greater than 50 mmHg despite maximum tolerable medical therapy are considered for invasive treatment. Intervention is recommended in patients with a mean Doppler gradient ≥30 mmHg or peak gradient ≥50 mmHg (class I, level of evidence C) [1]. The treatment options include surgical myomectomy if percutaneous alcohol ablation is ineffective. Medical therapy or septal ablation for presumed HCM would not have led to relief in symptoms in our case due to the fixed subaortic obstruction.

Another point worth mentioning is the presence of chordal rupture, which infrequently happens in HCM and usually occurs in elderly patients with the obstructive disease and involves the posterior leaflet more commonly than the anterior leaflet. Degeneration due to the myxomatous nature of the valve is the cause of the rupture in such cases and this correlates with the intraoperative findings in our case. The hypertrophied septum causes the flow to drag, and this pushes the mitral valve more anteriorly, leading to SAM and LVOT obstruction. The rupture of chordae tendineae causes the absence of the support of the AML, leading to the posterior shift of the coaptation with the alleviation of SAM and LVOT obstruction and improvement of symptoms. A review of the literature described 14 cases of hypertrophic obstructive cardiomyopathy (HOCM) with ruptured chordae tendineae [2]. These resulted in acute MR, and amelioration of the LVOT obstruction. This contrasts with the findings in our case, where chordae rupture was found with consistent SAM and LVOT gradient [3]. Four-dimensional CT can be used in such cases for the assessment of myocardial hypertrophy and the evaluation of exact mitral subvalvular abnormalities [4].

## Conclusions

We presented a clinical scenario where a SAM with mitral chordal rupture was misdiagnosed as HCM with SAM. This report highlights the need to differentiate between these two conditions through different imaging modalities as treatment strategies can differ based on the underlying etiology.
